# Clinical Spectrum, independent risk factors, and treatment outcomes of pediatric arrhythmias: a multicenter retrospective analysis in Xinjiang

**DOI:** 10.3389/fped.2026.1810814

**Published:** 2026-05-29

**Authors:** Jinyong Pan, Yan Zhang, Yan Guo, Hu Li, Fengling Zhang, Muqing Niu, Heyun Xiong, Cailing Chen, Hongbin Yang, Hua Guan, Yong Sun, Zhou Zhang, Jing Lv, Yonglin Chen

**Affiliations:** 1 The First Affiliated Hospital of Shihezi University, Shihezi, Xinjiang, China; 2 Department of Pediatrics, The First Affiliated Hospital of Shihezi University, Shihezi, Xinjiang, China; 3 Department of Pediatrics, First Division Hospital of Xinjiang Construction Corps, Alar, Xinjiang, China; 4 Department of Pediatrics, Second Division Hospital of Xinjiang Construction Corps, Tiemenguan, Xinjiang, China; 5 Department of Pediatrics, Fourth Division Hospital of Xinjiang Construction Corps, Kekedala, Xinjiang, China; 6 Department of Pediatrics, Seventh Division Hospital of Xinjiang Construction Corps, Huyanghe, Xinjiang, China; 7 Department of Pediatrics, 10th Division Hospital of Xinjiang Construction Corps, Beitun, Xinjiang, China; 8 Department of Pediatrics, Red Star Hospital, 13th Division, Hami, Xinjiang, China

**Keywords:** arrhythmia, catheter ablation, children, clinical characteristics, logistic regression, multicenter study, risk factors

## Abstract

**Background:**

Pediatric arrhythmias are heterogeneous and differ from adult disease in presentation and management; evidence from geographically vast, resource-variable regions of northwestern China remains limited, particularly in settings where access to pediatric electrophysiology services and prolonged rhythm monitoring is uneven.

**Objective:**

To describe the clinical spectrum of pediatric arrhythmias, identify independent risk factors, and assess treatment effectiveness and safety in a multicenter cohort from Xinjiang.

**Methods:**

This retrospective multicenter study included children (≤18 years) diagnosed with arrhythmia by ECG and/or 24-hour Holter monitoring in six prefecture-level hospitals in Xinjiang (January 2019 to December 2024). A 1:1 age- and sex-matched healthy cohort served as controls. Candidate factors were screened by univariable chi-square tests and entered into multivariable binary logistic regression; results are reported as ORs with 95% CIs. Management was categorized as regular follow-up, pharmacotherapy, or radiofrequency catheter ablation, with effectiveness assessed using standardized follow-up criteria.

**Results:**

We enrolled 232 children with arrhythmia (50.9% male), aged 1.2–18.0 years (mean 11.5 ± 3.6); 68.9% were ≥6 years. Supraventricular arrhythmias were most common (49.1%), and PSVT accounted for 35.3%. In multivariable analysis, congenital heart disease (OR 7.265, 95% CI 4.012–13.298), recent infection exposure (OR 6.452, 95% CI 3.605–11.521), and recurrent arrhythmia history (OR 8.216, 95% CI 4.458–15.169) were independent risk factors (all *P* < 0.05), while age ≥6 years was not. Management included follow-up (65.5%), pharmacotherapy (25.0%), and ablation (9.5%). Ablation in 22 PSVT patients achieved 100% acute success with no recurrence over 6–12 months. Among 102 patients with complete effectiveness evaluation, the overall response rate was 88.2%; four mild adverse drug reactions were observed.

**Conclusions:**

This Xinjiang multicenter cohort reveals region-specific divergences from published data: higher PSVT (35.3%) and ventricular arrhythmia (24.1%) proportions, and markedly lower ablation utilization (9.5%) vs. tertiary centers in eastern China and internationally. Congenital heart disease, infection exposure, and recurrence history were independent risk factors (ORs 6.5–8.2). Key actionable findings: (1) targeted ECG screening for high-risk children; (2) maintenance of school-based screening for subclinical detection; and (3) effective stratified management with pharmacotherapy and selective ablation, with regional capacity building needed to close the interventional care gap.

## 1 Introduction

Pediatric arrhythmias represent a common yet heterogeneous group of cardiovascular disorders characterized by diverse etiologies, age-specific clinical manifestations, and management paradigms that differ substantially from those in adults (1, 2). Their occurrence may be influenced by myocardial maturation, structural heart disease, inflammatory triggers, and external stressors (3, 4). Despite growing research interest, existing multicenter data in China are largely derived from developed eastern regions, where diagnostic and interventional resources are comparatively abundant (4, 5). In contrast, epidemiologic patterns, risk profiles, and real-world management outcomes from geographically vast and resource-variable settings remain underreported, particularly in regions where access to pediatric cardiology referral services, prolonged ambulatory ECG monitoring, and catheter-based electrophysiological treatment may vary across centers.

Xinjiang spans a wide geographic area, and pediatric healthcare capacity may vary across administrative divisions and referral networks. In this study, the participating centers were regional hospitals located in different divisions of the Xinjiang Production and Construction Corps healthcare system rather than a single uniform care tier. Consequently, the clinical characteristics and risk determinants of pediatric arrhythmias in this setting could demonstrate regional specificity influenced by healthcare access, environmental exposure, and referral patterns. We hypothesized that the clinical spectrum and management patterns of pediatric arrhythmias in Xinjiang would differ from those reported in more developed eastern regions of China and international cohorts, due to geographic dispersion, variable healthcare capacity across regional hospitals, and distinct referral pathways. Specifically, we anticipated a higher proportion of symptomatic or recurrent cases reflecting delayed diagnosis in dispersed populations, and lower utilization of interventional therapies such as catheter ablation reflecting resource constraints. This study therefore aimed to: (1) delineate the clinical spectrum and diagnostic patterns of pediatric arrhythmias in this underreported region; (2) identify high-risk and independent risk factors using univariable screening and multivariable logistic regression; and (3) evaluate effectiveness and safety of current stratified treatment strategies, with explicit comparison to national and international benchmarks to identify region-specific gaps and actionable targets for improvement (6, 7).

## 2 Methods

### 2.1 Study design and setting

This retrospective multicenter study included six regional hospitals located in different administrative divisions of Xinjiang. The study period spanned January 2019 to December 2024, with data locked on March 1, 2025, ensuring a minimum follow-up duration of 6 months for all included patients. During the study period, standard 12-lead ECG, inpatient bedside monitoring, and 24-hour Holter monitoring were routinely available across participating centers, whereas extended ambulatory monitoring modalities such as 14-day ECG patches, event recorders, wearable smart devices, and pediatric electrophysiological mapping resources were not uniformly available at all sites.

### 2.2 Participants

Arrhythmia group (cases): Children aged ≤18 years who met all of the following criteria were included: 1. Arrhythmia confirmed by standard ECG and/or 24-hour Holter monitoring, consistent with the 2020 pediatric arrhythmia diagnostic and treatment guideline used clinically in the participating centers (8, 9). 2. Complete clinical records including demographics, diagnostic evidence, symptoms, etiologic evaluations, treatment strategy, and follow-up data for ≥6 months. 3. First diagnosis or major clinical management occurred at one of the participating hospitals.

Exclusion criteria: 1. Transient arrhythmias secondary to reversible causes (e.g., electrolyte disturbances, medication-related effects, perioperative stress) (10). 2. Severe comorbidities (e.g., malignancy, severe hepatic/renal failure, severe structural neurologic disease). 3. Missing ≥2 key variables required for etiologic assessment or outcome evaluation. 4. Follow-up duration <6 months.

Control group: Healthy children undergoing routine pediatric checkups during the same period and from the same six hospitals were matched 1:1 to cases by exact age (±6 months) and sex. Controls were recruited from the same six hospitals during the identical study period (January 2019 to December 2024) and from the same geographic catchment areas as cases to minimize regional and socioeconomic confounding. Matching procedure: For each case, potential controls meeting age and sex criteria were identified from the same hospital's pediatric checkup registry; the control with the closest birth date was selected. If no suitable control was available at the same hospital, the next nearest hospital within the same administrative division was used [affecting 12 controls (5.2%)]. Controls had no known arrhythmia or structural heart disease, no acute infection within 2 weeks of assessment, normal ECG, and myocardial enzyme tests (11, 12). Additional matching variables (e.g., socioeconomic status, ethnicity) were not included due to incomplete data capture in retrospective records; however, cases and controls were drawn from the same catchment areas with homogeneous socioeconomic profiles (predominantly agricultural and urban-worker families within the Xinjiang Production and Construction Corps system), minimizing residual confounding. The 1:1 age- and sex-matched control group was included specifically to enable rigorous risk factor analysis. Without a matched comparison group from the same source population, it would not have been possible to distinguish factors independently associated with arrhythmia from those that are simply common in the general pediatric population, or to calculate adjusted odds ratios.

### 2.3 Data collection and variables

Clinical data were extracted from electronic medical records and Xinjiang regional collaboration platform into a standardized database, including: 1. Demographics and care metrics: sex, age, hospitalizations, length of stay, and follow-up duration. 2. Clinical features: symptoms, episodic pattern, recurrence history, infection exposure history, and congenital heart disease history. Recent infection exposure was defined as a clinically documented acute infection diagnosed within 4 weeks (28 days) prior to arrhythmia onset or diagnosis, based on the following standardized criteria: (1) time window: 4 weeks preceding the index arrhythmia episode or hospital admission for arrhythmia diagnosis; (2) diagnostic confirmation: infection documented by physician diagnosis in medical records with supporting evidence (fever ≥38 °C recorded on ≥2 occasions, abnormal white blood cell count, or positive microbiological/rapid antigen test where performed); self-reported symptoms without clinical documentation were excluded; (3) infection type: predominantly upper respiratory tract infections (pharyngitis, tonsillitis, bronchitis) and pneumonia (viral or bacterial); gastrointestinal and urinary tract infections were included if clinically confirmed but comprised <5% of cases; (4) exclusions: chronic infections, subclinical carrier states, and perioperative infections were excluded. This 4-week window was selected based on the established inflammatory myocardial vulnerability period following acute infection. 3. Diagnostic indicators: diagnostic modality (ECG/Holter), arrhythmia subtype, myocardial enzymes (e.g., CK-MB, LDH, HBDH where available) (13, 14). 4. Treatment and outcomes: follow-up only, pharmacotherapy (drug type and regimen), catheter ablation details, effectiveness assessment, and adverse events.

### 2.4 Outcomes and effectiveness evaluation

Effectiveness analyses were restricted to children who underwent active treatment or structured serial reassessment for clinically relevant arrhythmias; isolated asymptomatic first-degree atrioventricular block managed with observation alone was not included in treatment-effectiveness comparisons. Of 232 cases, 130 were excluded from effectiveness evaluation for the following reasons: 65 (28.0%) were managed with observation alone (including 4 isolated first-degree AV block), 38 (16.4%) had follow-up <6 months due to transfer to other facilities or loss to follow-up, 20 (8.6%) had incomplete Holter or symptom documentation, and 7 (3.0%) declined active monitoring. Effectiveness was assessed using standardized criteria based on pediatric arrhythmia effectiveness evaluation principles and the cohort's follow-up structure (8, 15): 1. Markedly effective: complete symptom resolution; arrhythmia terminated; Holter normal; no recurrence for 6 months. 2. Effective: symptom improvement; ≥70% reduction in attack frequency and/or ≥50% reduction in arrhythmia burden on Holter; no severe episodes during follow-up. 3. Ineffective: no improvement or worsening; <50% reduction in attack frequency or need for treatment escalation/referral.

Overall response rate = (markedly effective + effective)/total evaluated × 100%.

### 2.5 Statistical analysis

Analyses were performed using SPSS (version 26.0). Continuous variables were summarized as mean ± SD (or median with interquartile range if non-normal), and categorical variables as counts and percentages. Univariable *χ*^2^-tests screened candidate risk factors with arrhythmia occurrence as the dependent variable. Variables with *P* < 0.05 were entered into a multivariable binary logistic regression model (enter method). Multicollinearity was evaluated using variance inflation factors (VIF) and pairwise correlation matrices; VIF > 10 or correlation coefficients |*r*| > 0.8 were considered indicative of significant collinearity. Model discrimination was assessed using the area under the receiver operating characteristic curve (AUC) with 95% confidence intervals. Model calibration was assessed using Hosmer–Lemeshow goodness-of-fit test (grouped into 10 deciles) and visual inspection of calibration plots (observed vs. predicted probabilities). A two-sided *P* < 0.05 was considered statistically significant (16).

## 3 Results

### 3.1 Participant characteristics

Among 232 children with arrhythmia, sex distribution was balanced [male 118 [50.9%], female 114 [49.1%]]. Age ranged from 1.2 to 18.0 years (mean 11.5 ± 3.6). School-aged children and adolescents predominated, with 160 (68.9%) aged ≥6 years. The mean length of hospital stay was 6.2 ± 3.1 days, with most children hospitalized once (75.9%). Follow-up duration ranged from 6 to 24 months (mean 11.5 ± 4.2), with a follow-up completion rate of 89.7%.

### 3.2 Clinical presentation

Most children were symptomatic (182/232, 78.4%). Common symptoms included palpitations/chest tightness (50.5%), chest pain (15.4%), dizziness (12.1%), and perceived heart rate abnormalities (16.5%). Notably, 50 children (21.6%) were asymptomatic; these cases were detected during school examinations or incidentally during visits for other conditions, highlighting the potential for subclinical arrhythmias (17, 18). School-based ECG screening programs have demonstrated capacity to detect previously unrecognized conduction abnormalities and pre-excitation syndromes in pediatric populations (18, 19).

### 3.3 Diagnostic modalities

Standard ECG established diagnosis in 148 children (63.8%), while 24-hour Holter monitoring was required in 84 (36.2%), predominantly for paroxysmal arrhythmias (13, 14). The high yield of screening ECG in asymptomatic children supports broader implementation of systematic pediatric electrocardiography evaluation (19).

### 3.4 Arrhythmia spectrum

Supraventricular arrhythmias accounted for 49.1% (114/232), with PSVT representing the most frequent subtype (82/232, 35.3%). Ventricular arrhythmias comprised 24.1%, sinus rhythm-related arrhythmias 17.7%, junctional arrhythmias 6.9%, and conduction block/mixed types 3.9% (1, 2)(see Table 1).

**Table 1:** Arrhythmia categories and subtypes among children with arrhythmia (*n* = 232).

**Arrhythmia category and subtype**	**No. (%)**
Supraventricular arrhythmias	**114 (49.1)**
Paroxysmal supraventricular tachycardia (PSVT)	82 (35.3)
Atrial premature contractions	24 (10.3)
Atrial tachycardia	8 (3.4)
Ventricular arrhythmias	**56 (24.1)**
Ventricular premature contractions	42 (18.1)
Ventricular tachycardia	14 (6.0)
Sinus rhythm–related arrhythmias	**41 (17.7)**
Sinus tachycardia	22 (9.5)
Sinus arrhythmia	19 (8.2)
Atrioventricular junctional arrhythmias	**16 (6.9)**
Junctional tachycardia	10 (4.3)
Junctional premature contractions	6 (2.6)
Conduction block and mixed types	**9 (3.9)**
First-degree atrioventricular block	4 (1.7)
Mixed arrhythmias	5 (2.2)

PSVT, paroxysmal supraventricular tachycardia.

Bold values indicate arrhythmia categories; non-bold rows indicate corresponding subtypes. Percentages are calculated using the total cohort (*n* = 232) as the denominator. Some children with mixed arrhythmias may be counted in more than one category; therefore, counts across categories may not sum exactly to the total cohort. Isolated asymptomatic first-degree atrioventricular block was retained in the descriptive spectrum table but excluded from treatment-effectiveness comparisons.

### 3.5 Etiology and triggers (descriptive)

Congenital heart disease was present in 42 children (18.1%), most commonly atrial septal defects. Detailed lesion-by-arrhythmia pairing was incompletely available in a subset of transferred multicenter records and therefore is not presented quantitatively. Recent infection exposure (documented within 4 weeks prior to arrhythmia onset) was documented in 48 children (20.7%), predominantly upper respiratory tract infections (clinically confirmed). A recurrent arrhythmia history was noted in 68 (29.3%).

### 3.6 Risk factor analyses

In univariable analyses, age ≥6 years, congenital heart disease history, infection exposure, and recurrent arrhythmia history were associated with arrhythmia occurrence (all *P* < 0.05). In multivariable logistic regression, congenital heart disease history (OR 7.265, 95% CI 4.012–13.298), infection exposure (OR 6.452, 95% CI 3.605–11.521), and recurrent arrhythmia history (OR 8.216, 95% CI 4.458–15.169) were independent risk factors (all *P* < 0.001). Age ≥6 years was not independently associated after adjustment. Multicollinearity diagnostics showed no significant correlation among predictors: all VIF < 1.5 (range 1.1–1.4) and all pairwise |*r*| < 0.3, indicating absence of multicollinearity. The model showed good discrimination with an AUC of 0.826 (95% CI: 0.778–0.874). Model fit was acceptable (Hosmer–Lemeshow *χ*^2^ = 4.215, *P* = 0.836) (11, 12)(see Table 2).

**Table 2:** Independent risk factors for pediatric arrhythmia: multivariable binary logistic regression.

**Variable**	**Adjusted OR (95% CI)**	***P* value**
History of congenital heart disease	7.265 (4.012–13.298)	<0.001
History of infection exposure	6.452 (3.605–11.521)	<0.001
History of recurrent arrhythmia episodes	8.216 (4.458–15.169)	<0.001
Age ≥ 6 years	1.243 (0.725–2.136)	0.444

CI, confidence interval; OR, odds ratio.

Model specification: Binary logistic regression (enter method).

Reference categories: No congenital heart disease history; no infection exposure history; no recurrent arrhythmia history; age <6 years.

Model fit: Hosmer–Lemeshow test *χ*^2^ = 4.215, *P* = 0.836.

### 3.7 Treatment patterns and outcomes

A stratified approach was adopted: regular follow-up (152/232, 65.5%), pharmacotherapy (58/232, 25.0%), and radiofrequency catheter ablation (22/232, 9.5%). Pharmacotherapy commonly included propafenone, with other agents used selectively (15, 18, 23). Ablation was performed exclusively in PSVT patients (age 10–15 years) with 100% acute procedural success and no recurrence during 6–12 months follow-up (*n* = 22) (20, 21). These findings should be interpreted cautiously given the small sample size, single-operator experience at two centers, and limited follow-up duration. For context, published pediatric PSVT ablation series report acute success rates of 95%–98% and 1-year recurrence rates of 2%–5% in larger cohorts from high-volume centers.

Missing effectiveness data. Of 232 cases, 130 were excluded from effectiveness evaluation: 65 (28.0%) were managed with observation alone (including 4 isolated first-degree AV block), 38 (16.4%) had follow-up <6 months due to transfer to other facilities or loss to follow-up, 20 (8.6%) had incomplete Holter or symptom documentation, and 7 (3.0%) declined active monitoring. Baseline characteristics were comparable between evaluated (*n* = 102) and non-evaluated (*n* = 130) children (Supplementary Table S1): age (11.2 ± 3.4 vs. 11.8 ± 3.7 years, *P* = 0.21), sex (male 51.0% vs. 50.8%, *P* = 0.97), congenital heart disease (17.6% vs. 18.5%, *P* = 0.85), infection exposure (19.6% vs. 21.5%, *P* = 0.72), and arrhythmia subtype distribution (*P* = 0.68). These similarities suggest that attrition bias is unlikely to substantially affect treatment-effectiveness conclusions. Among 102 children with complete effectiveness evaluation, 32 (31.4%) were markedly effective, 58 (56.9%) effective, and 12 (11.8%) ineffective, yielding an overall response rate of 88.2%. Only four mild gastrointestinal adverse drug reactions were observed; no severe drug events or procedure-related complications occurred (15, 21). The markedly effective subgroup consisted of children with complete symptom resolution, arrhythmia termination, and no recurrence during the prespecified follow-up window; this subgroup is reported separately to provide greater clinical granularity.

### 3.8 Comparison with national and international data

To contextualize our findings, we compared key metrics with published multicenter data from eastern China and international cohorts (Table 3). The proportion of PSVT in our cohort (35.3%) was higher than reported ranges in eastern Chinese tertiary centers (20%–30%) and North American pediatric networks (25%–35%), while ventricular arrhythmias (24.1%) exceeded published ranges in both settings (15%–20% and 15%–22%, respectively). Conversely, the utilization of radiofrequency catheter ablation in our cohort (9.5%) was substantially lower than in eastern Chinese tertiary centers (>35%) and North American pediatric electrophysiology networks (45%–55%). The proportion of asymptomatic cases detected incidentally (21.6%) was higher than in urban Chinese cohorts (12%–15%), likely reflecting school-based screening programs in our catchment areas.

**Table 3:** Comparison of pediatric arrhythmia characteristics across regions.

Characteristic	Present study (Xinjiang)	Eastern China [4,5]	North America [19,20]
Study period	2019–2024	2015–2022	2010–2018
Centers	6 regional hospitals	3–5 tertiary centers	12–15 specialized centers
Sample size	232	450–680	800–1,200
Age, mean ± SD (years)	11.5 ± 3.6	9.8 ± 4.2	10.2 ± 5.1
Male, %	50.9	52–55	51–54
Supraventricular arrhythmias, %	49.1	45–52	48–55
PSVT, %	35.3	20–30	25–35
Ventricular arrhythmias, %	24.1	15–20	15–22
Sinus rhythm-related, %	17.7	15–22	12–18
Conduction block/mixed, %	3.9	4–7	3–6
Congenital heart disease, %	18.1	10–15	10–14
Asymptomatic detection, %	21.6	10–15	12–18
Catheter ablation rate, %	9.5	30–45	40–55
Acute ablation success, %	100 (*n* = 22)	95–98	95–98

Values for eastern Chinese tertiary centers and North American pediatric networks represent approximate ranges derived from published single-center series, narrative reviews, and consensus statements, as no directly comparable prospective multicenter pediatric arrhythmia registries exist. Bold values indicate notable divergences from the present cohort. PSVT, paroxysmal supraventricular tachycardia.

These comparisons suggest that our cohort was enriched with more symptomatic, recurrent, or structurally complex cases—consistent with a referral and diagnostic environment where extended ambulatory monitoring and primary care electrocardiography screening are less uniformly available.

## 4 Discussion

This multicenter study provides the first real-world clinical data on pediatric arrhythmias across six regional hospitals in Xinjiang, northwestern China. Compared with published data from more developed regions, our cohort showed distinctive patterns in arrhythmia spectrum and management intensity that reflect the interaction between disease biology and healthcare system architecture. As a geographically extensive and resource-variable region with limited prior epidemiologic evidence, our findings fill an important evidence gap that complements existing data from more developed eastern regions of China. In this cohort, pediatric arrhythmias occurred predominantly in school-aged children and adolescents, with supraventricular arrhythmias—particularly paroxysmal supraventricular tachycardia (PSVT)—representing the most common subtype. A considerable proportion of cases were asymptomatic and identified incidentally, supporting the need for heightened screening awareness in high-risk groups (1, 17).

After adjusting for confounders, three independent risk factors emerged: congenital heart disease history, infection exposure, and recurrent arrhythmia history, each associated with markedly increased odds of arrhythmia (ORs > 6). Recurrent arrhythmia history showed the strongest association, suggesting the importance of individualized long-term follow-up and proactive rhythm monitoring in children with prior episodes (11). Congenital heart disease, especially atrial septal defects in this dataset, likely provides a structural substrate for arrhythmogenesis (2, 3). Infection exposure—predominantly respiratory—may contribute through inflammatory myocardial involvement and autonomic imbalance; clinicians should maintain vigilance for arrhythmias during pediatric infectious episodes, particularly when symptoms such as palpitations or chest discomfort are present (3, 24). However, reverse causality is a significant limitation: children presenting with arrhythmia symptoms undergo more intensive clinical evaluation, including infection screening, than healthy children undergoing routine checkups, potentially inflating the observed association. Future prospective studies with standardized infection surveillance and sensitivity analyses excluding concurrently diagnosed infections are needed to clarify temporal relationships and assess the true causal contribution. Age ≥6 years was not independently associated after adjustment.

Heterogeneity of arrhythmia mechanisms. This study pooled multiple arrhythmia subtypes with divergent electrophysiological mechanisms. The identified risk factors likely operate differently across categories: CHD provides structural substrates for both supraventricular and ventricular arrhythmias, whereas infection-related autonomic imbalance may preferentially trigger supraventricular tachycardia. Subgroup analyses were not performed due to insufficient power in individual subgroups (e.g., only 14 ventricular tachycardia cases). Future studies with larger samples should model risk factors separately for PSVT, ventricular arrhythmias, and conduction disorders.

Regional divergence and its mechanisms. Three distinctive features of our cohort, when compared with published data from eastern Chinese tertiary centers and international networks, warrant mechanistic interpretation. First, the higher proportion of PSVT (35.3% vs. approximately 20%–30% in eastern Chinese tertiary centers and 25%–35% in North American networks) likely reflects a diagnostic and referral filter: in Xinjiang's geographically dispersed population, children with brief or mildly symptomatic episodes may not reach regional hospitals, whereas those with persistent, recurrent, or hemodynamically significant PSVT are selectively referred. This is supported by the 29.3% recurrence history and the 36.2% Holter utilization rate in our cohort. Second, the elevated ventricular arrhythmia rate (24.1% vs. 15%–20%) correlates with the higher burden of congenital heart disease (18.1%), predominantly atrial septal defects, which provide structural substrates for arrhythmogenesis. Third, the markedly lower catheter ablation rate (9.5% vs. >35%–55%) does not indicate lower disease severity but rather supply-side constraints: pediatric electrophysiology expertise and equipment were available at limited centers during the study period, and geographic distance between administrative divisions poses practical barriers to referral. Conversely, the 21.6% asymptomatic detection rate reflects school-based screening programs in our catchment areas, which identify subclinical cases that might otherwise remain undetected.

The observed stratified strategy—follow-up for mild/stable cases, pharmacotherapy for symptomatic/clinically significant arrhythmias, and ablation for refractory PSVT—was associated with high overall effectiveness and favorable safety. Propafenone was commonly used and appeared clinically effective in this real-world setting. Catheter ablation showed excellent outcomes for PSVT, supporting its role for refractory or recurrent cases when resources and expertise allow (20, 21). The 100% acute success and zero recurrence in our 22 patients are encouraging but must be interpreted with caution: the sample is small, follow-up is limited to 6–12 months, and outcomes may not generalize to lower-volume centers. These results align with published benchmarks from prospective multicenter registries (acute success 95%–96%, 1-year recurrence 4%–8%) (20, 22) but require validation in larger cohorts with longer follow-up. To avoid distortion of the treatment-effectiveness results, isolated asymptomatic first-degree atrioventricular block was not considered part of the comparative effectiveness set.

Clinical and policy implications. These findings translate into three actionable strategies for Xinjiang and comparable resource-variable settings. First, targeted screening: children with congenital heart disease or recent infection within 4 weeks should undergo routine ECG evaluation, given their substantially elevated adjusted odds (ORs 6.5–8.2). School-based screening programs, which detected 21.6% of cases in our cohort incidentally, should be maintained and expanded (18, 19). Second, diagnostic capacity building: investment in extended ambulatory monitoring (14-day patch monitors, event recorders) at regional hospitals could reduce diagnostic delay and unnecessary referrals. Third, interventional access expansion: the ablation rate of 9.5% represents a substantial treatment gap. Establishing a pediatric electrophysiology referral network with centralized ablation hubs and telemedicine-supported pre-procedure evaluation could improve access without requiring full infrastructure at every center. Propafenone, effective and well-tolerated in our pharmacotherapy group, may serve as practical bridge therapy for symptomatic patients awaiting ablation or residing in remote areas.

This study has several limitations inherent to retrospective designs, including potential selection bias and incomplete capture of cases managed at primary facilities or referred directly to tertiary centers. Age distribution may reflect regional healthcare access and referral patterns. The high proportion of ventricular arrhythmias (24.1%) should be interpreted cautiously, as our cohort comprised hospitalized and referred children with a high prevalence of congenital heart disease and symptomatic arrhythmias, rather than a general population sample. In addition, subgroup analyses by arrhythmia subtype were not performed, limiting inference regarding factor-specific effects across different arrhythmia categories. Detailed congenital heart disease lesion-arrhythmia pairings were not uniformly available across all centers, which limited more granular subtype-specific analyses. In addition, the retrospective database did not support a robust separate effectiveness analysis for each arrhythmia subtype. Finally, effectiveness evaluation was available for a subset of patients (102/232, 44.0%), and missing effectiveness data may introduce attrition bias; prospective studies with standardized follow-up protocols are warranted (5, 7, 16).

These region-specific findings highlight actionable targets for improving equity in pediatric arrhythmia care in geographically dispersed, resource-variable settings. Despite these limitations, this study provides important region-specific data for a historically understudied population. The findings reflect real-world clinical practice in regional hospitals and may be generalizable to similar resource-variable settings worldwide. Future work should include prospective multicenter enrollment with broader inclusion of infants, subtype-specific modeling of risk factors, harmonized outcome definitions, and strengthened regional referral networks and interventional training programs to expand access to catheter ablation for complex or refractory arrhythmias (6, 22).

## 5 Conclusions

This multicenter study from Xinjiang, northwestern China, reveals region-specific patterns in pediatric arrhythmia epidemiology and management that diverge from published data in more developed regions: a higher proportion of PSVT and ventricular arrhythmias, reflecting referral patterns and a higher burden of congenital heart disease; and markedly lower utilization of catheter ablation (9.5%), reflecting resource constraints. Three clinical messages emerge: (1) children with congenital heart disease or recent infection warrant proactive ECG screening, given their substantially elevated risk (ORs 6.5–8.2); (2) school-based and primary care screening effectively detect subclinical cases and should be expanded; and (3) stratified management with pharmacotherapy (including propafenone) and selective ablation is effective, but closing the interventional gap through regional capacity building and referral networks represents an urgent unmet need for geographically dispersed populations. These findings provide evidence-based targets for improving equity in pediatric arrhythmia care in resource-variable settings.

## Data Availability

The raw data supporting the conclusions of this article will be made available by the authors, without undue reservation.

## Supplementary material

Supplementary Table S1 Baseline characteristics of children with complete effectiveness evaluation and those excluded from effectiveness evaluation.
