# Supplementary material for: Clinical Spectrum, independent risk factors, and treatment outcomes of pediatric arrhythmias: a multicenter retrospective analysis in Xinjiang
Source: Front Pediatr. 2026 May 29;14:1810814. doi: 10.3389/fped.2026.1810814 (PMC13260524; doi:10.3389/fped.2026.1810814)
Supplement: Supplementary Table S1 — Baseline characteristics of children with complete effectiveness evaluation and those excluded from effectiveness evaluation. [file Table1.docx]

**Supplementary Table S1**

**Baseline characteristics of children with complete effectiveness evaluation and those excluded from effectiveness evaluation**

*Clinical Spectrum, Independent Risk Factors, and Treatment Outcomes of Pediatric Arrhythmias: A Multicenter Retrospective Analysis in Xinjiang*

| **Characteristic** | **Complete effectiveness evaluation (n = 102)** | **Excluded from effectiveness evaluation (n = 130)** | **P value** |
| --- | --- | --- | --- |
| Age, years, mean ± SD | 11.2 ± 3.4 | 11.8 ± 3.7 | 0.21 |
| Male sex, n (%) | 52 (51.0) | 66 (50.8) | 0.97 |
| Congenital heart disease, n (%) | 18 (17.6) | 24 (18.5) | 0.85 |
| Recent infection exposure, n (%) | 20 (19.6) | 28 (21.5) | 0.72 |
| Arrhythmia subtype distribution | Overall categorical distribution compared between groups | Overall categorical distribution compared between groups | 0.68 |

Values are presented as mean ± SD or n (%), unless otherwise indicated.

P values correspond to comparisons between children with complete effectiveness evaluation and those excluded from effectiveness evaluation.

For arrhythmia subtype distribution, the overall categorical distribution was compared between groups.

SD, standard deviation.
